# Insights into SARS-CoV-2, the Coronavirus Underlying COVID-19: Recent Genomic Data and the Development of Reverse Genetics Systems

**DOI:** 10.1099/jgv.0.001458

**Published:** 2020-06-24

**Authors:** Severino Jefferson Ribeiro da Silva, Renata Pessôa Germano Mendes, Caroline Targino Alves da Silva, Alessio Lorusso, Alain Kohl, Lindomar Pena

**Affiliations:** ^1^​ Department of Virology, Aggeu Magalhães Institute (IAM), Oswaldo Cruz Foundation (Fiocruz), 50670-420, Recife Pernambuco, Brazil; ^2^​ Istituto Zooprofilattico Sperimentale dell’Abruzzo e del Molise (IZSAM), Teramo, Italy; ^3^​ MRC-University of Glasgow Centre for Virus Research, Glasgow, Scotland G61 1QH, UK

**Keywords:** SARS-CoV-2, reverse genetics, COVID-19, genomics

## Abstract

The emergence and rapid worldwide spread of a novel pandemic of acute respiratory disease – eventually named coronavirus disease 2019 (COVID-19) by the World Health Organization (WHO) – across the human population has raised great concerns. It prompted a mobilization around the globe to study the underlying pathogen, a close relative of severe acute respiratory syndrome coronavirus (SARS-CoV) called severe acute respiratory syndrome coronavirus 2 (SARS-CoV-2). Numerous genome sequences of SARS-CoV-2 are now available and in-depth analyses are advancing. These will allow detailed characterization of sequence and protein functions, including comparative studies. Care should be taken when inferring function from sequence information alone, and reverse genetics systems can be used to unequivocally identify key features. For example, the molecular markers of virulence, host range and transmissibility of SARS-CoV-2 can be compared to those of related viruses in order to shed light on the biology of this emerging pathogen. Here, we summarize some recent insights from genomic studies and strategies for reverse genetics systems to generate recombinant viruses, which will be useful to investigate viral genome properties and evolution.

Coronavirus disease 2019 (COVID-19) [1] is caused by severe acute respiratory syndrome coronavirus 2 (SARS-CoV-2), a recently identified virus (December 2019, Wuhan, Hubei Province, China) [2, 3] belonging to the family *Coronaviridae*, subfamily *Coronavirinae,* genus *Betacoronavirus*, in the species *severe acute respiratory syndrome-related coronavirus* [4]. This pandemic has led to disease and mortality in populations across the globe [1, 5]. On 11 March 2020, the World Health Organization (WHO) declared a SARS-CoV-2 pandemic. As of 31 May 2020, more than 6 million COVID-19 cases have been reported in 188 countries, resulting in 370 000 deaths worldwide. In addition to the genus *Betacoronavirus*, the subfamily *Coronavirinae* is composed of the genera *Alphacoronavirus*, *Gammacoronavirus* and *Deltacoronavirus*. Seven types of CoVs are known to cause human disease. Several alphacoronaviruses (HC 229E and HC NL63) and betacoronaviruses (HC OC43 and HC HKU1) are endemic and cause mild respiratory tract infections [6, 7]. Previous to SARS-CoV-2, two betacoronaviruses had already emerged in the 21st century causing severe respiratory disease: SARS-CoV (same species as SARS-CoV-2) and Middle East respiratory syndrome CoV (MERS-CoV), allowing comparisons to be made between these three viruses and their respective diseases [8–10]. In January 2020 the complete viral genome sequences from five patients in Wuhan during an early stage of the outbreak were published and SARS-CoV-2 was found to be a novel CoV, with just under 80 % sequence identity to SARS-CoV [2]. The viruses most closely related to SARS-CoV-2 were coronaviruses isolated from bats, in particular RaTG13 [2, 11]. Therefore, it was hypothesized that bats – a known reservoir of coronaviruses – could serve as a reservoir for this novel coronavirus. However, it is not clear whether transmission of SARS-CoV-2 to humans occurred directly from bats or through an intermediate host [11–16]. The genome sequences of numerous SARS-CoV-2 strains from across the globe are now publicly available.

Viral replication takes place in the cytoplasm. The genome organization of the virus is summarized in Fig. 1 (alongside descriptions of reverse genetics systems; see below), with the overall structure and key elements of the genome being comparable to those of related coronaviruses. Viral replicase activities are directed by 16 non-structural proteins that are produced following the proteolytic cleavage of 2 replicase polyproteins [open reading frames (ORFs) 1a/b]. The genome also encodes the structural proteins N, S, E and M, as well as accessory proteins [17–24]. The genome termini play critical roles in replication and transcription; ORFs 1a/b are translated from the genome, but subgenomic mRNAs mediate the expression of the remaining viral proteins [25, 26].

**Fig. 1.**
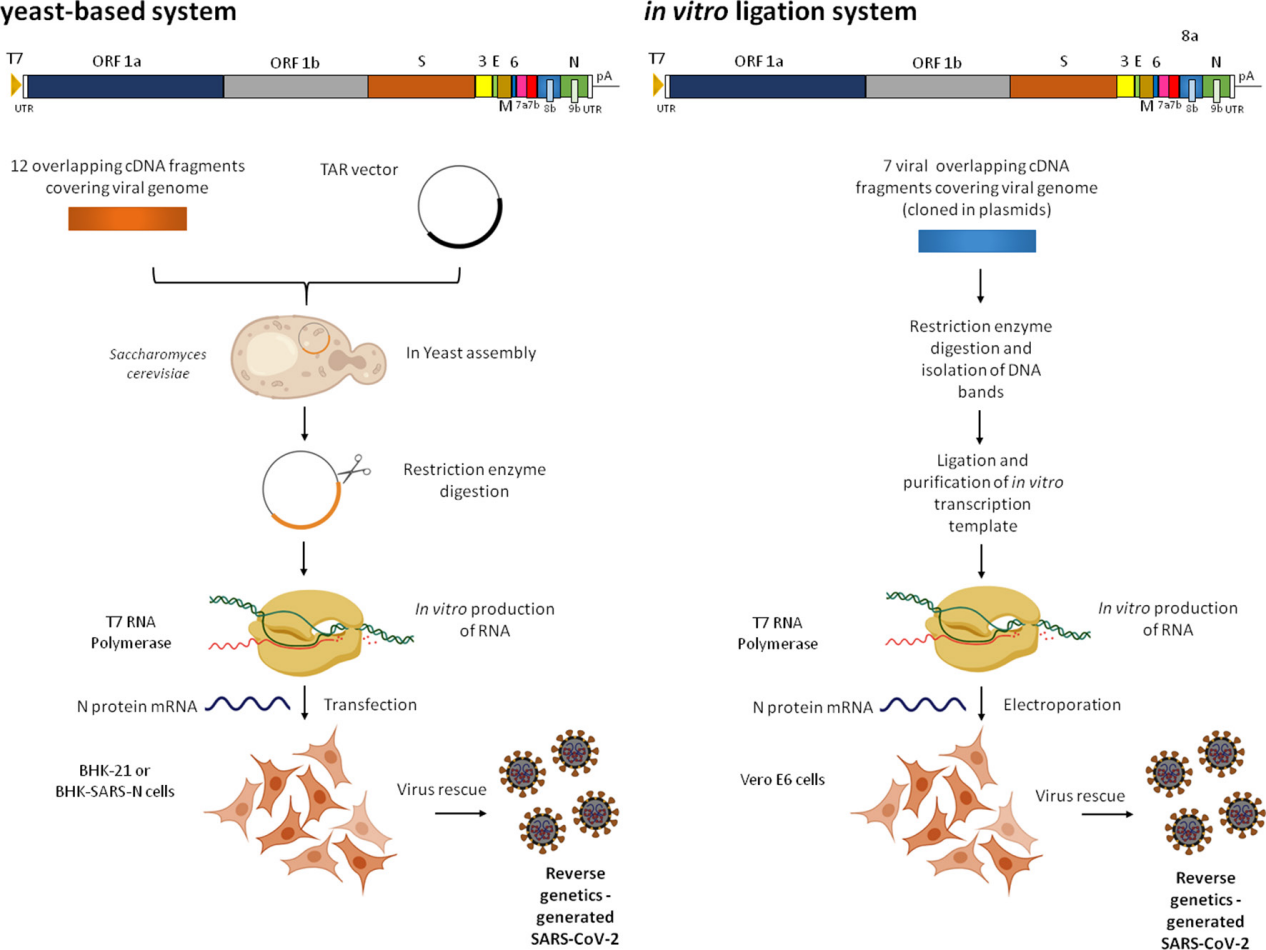
Reverse genetics systems for SARS-CoV-2. Viral genome and organization are shown at the top of each panel. (a) Description of yeast-based assembly and rescue system. Twelve viral subgenomic cDNA fragments were assembled in *Saccharomyces cerevisiae* using transformation-associated recombination (TAR) cloning to maintain the genome as a yeast artificial chromosome (YAC). *In vitro*-transcribed (by T7 RNA polymerase) viral genome RNAs were electroporated into BHK-21 cells (or BHK-SARS-N) together with an mRNA encoding the SARS-CoV-2 N protein to rescue viable virus. (b) Description of *in vitro* ligation system. In this approach, seven contiguous cDNA fragments covering the entire viral genome were isolated from plasmid vectors and directionally ligated to assemble the full-length viral genome. *In vitro*-transcribed (by T7 RNA polymerase) genome RNA was transfected into Vero E6 cells along with mRNA encoding N protein to recover infectious SARS-CoV-2. A schematic representation of the SARS-CoV-2 genome organization is shown in the upper part of the panels. T7, T7 RNA polymerase promoter; UTR, untranslated region; pA, poly (A) tail. Created with Biorender.com

Clearly COVID-19 is different from the disease caused by SARS-CoV-2’s close relative, SARS-CoV. Indeed the case fatality rates for COVID-19 are lower, and the disease can be mild or asymptomatic [27]. Investigating the differences between the two related viruses is thus of critical importance for future investigations. Analysis of SARS-CoV-2 sequences showed that the spike protein (S) has a furin(-like) cleavage site that is absent in related coronaviruses, and this was experimentally confirmed [28, 29]. Intriguingly, the loss of this cleavage site was shown upon passaging the virus in cell culture [30]. Processing by furin cleavage could have implications for virulence and/or adaptation. Moreover, roles for nonstructural proteins nsp2, nsp3, ns7b and ns8 in the pathogenesis of SARS-CoV-2 have been proposed following analysis of selective pressure on ORFs [31, 32]. Recent analysis of over 200 SARS-CoV-2 sequences classified the virus into 5 main groups based on high-frequency mutations (mutant allele frequency >5 %), with group 4 most frequently being found outside mainland China. Intriguingly, analysis of group 4 SARS-CoV-2 genome data from France showed that viruses carrying a mutation in 3a also often have a mutation in S (ORF3a:c.752gGt>gTt and S:c.1099Gtc>Ttc), although the biological relevance of this is not clear [33]. Moreover, comparative analysis between SARS-CoV-2 and RaTG13 has suggested some evidence of limited positive selection, although this cannot be interpreted as evidence for adaptation to humans [11]. More recently, it has been proposed that the D614G mutation in the S glycoprotein increases the transmissibility of SARS-CoV-2, as evidenced by sequence analysis [34]. However, care should be taken when inferring function from sequence information alone. In the context of another respiratory virus, influenza A virus (IAV), the PB1-F2 protein was identified as an important virulence factor and the N66S mutation in this protein was associated with the high lethality of the 1918 and other IAVs. However, reverse genetics studies found that the role of PB1-F2 (and the N66S polymorphism) in the virulence of different IAVs was host- and strain-dependent, ranging from increased virulence to no effect and even attenuation [35, 36]. Together, these examples illustrate how phenotype inference from sequence information needs experimental confirmation – importantly, in relevant systems.

Reverse genetics is a powerful technique for the generation of an infectious virus from the cloned full-length cDNA/synthetized DNA of a given virus. Manipulation of the DNA by well-established molecular biology methods allows modification of the sequence before virus production, if this is desired. It allows unequivocal identification of molecular markers for a given virus, including the genome features, virulence, host range, etc. of a given virus, and as such it is a key component in the study of coronaviruses [37]. Ultimately, such systems will be required to answer questions on various aspects of SARS-CoV-2 biology and genomics. The generation of coronaviruses entirely from full-length DNA can be challenging. This is mainly because of the large genome size of coronaviruses (~30 Kb) as well as the instability of genome sequences during cloning. However, such systems, as well as minireplicons (replication/transcription active, propagation-incompetent viral RNAs), have been developed successfully in the past for coronaviruses, including SARS-CoV. This was achieved through the use of bacterial artificial chromosomes, *in vitro* ligation of DNA fragments covering the full-length genome, or vaccinia virus-based expression vectors [38]. The systems included reverse genetics for SARS-CoV as well as MERS-CoV [39–43]. A ligation-based methodology combining individual stretches of DNA covering the SARS-CoV-2 genome (originally cloned into plasmids), followed by T7 RNA polymerase-based transcription to produce viral RNA, was used successfully to recover this virus [44]. Similarly, a yeast artificial chromosome-based system was developed recently to propagate the full-length SARS-CoV-2 genome assembled from DNA. Again, viral RNA was transcribed by T7 RNA polymerase before transfection [45]. Both of these recent methodologies are summarized in Fig. 1. An mRNA encoding the SARS-CoV-2 N protein was co-transfected in both approaches to enhance the infectivity of viral RNA transcripts. Both approaches allowed the successful generation of fluorescent protein-expressing recombinant viruses. The availability of SARS-CoV-2 reverse genetics systems will allow effective manipulation of its genome and unravel questions over entry, gene expression, replication, tropism, etc highlighted by genomic analysis as discussed above. These technologies can also be applied to the generation of vaccine candidates and the discovery of antivirals against this devastating human pathogen. The rapid development of such systems for SARS-CoV-2 using different approaches is testimony to the pioneering work carried out with related viruses and underlines the need for continuous research on these and other pathogens. They will greatly enhance our ability to investigate this novel pathogen, as well as coronaviruses that may emerge in the future.
